# Progression From Isolated Posterior Pituitary Dysfunction to Combined Anterior Hormone Deficiencies With Pituitary Stalk Enlargement in Suspected Lymphocytic Hypophysitis: A Case Report

**DOI:** 10.7759/cureus.98595

**Published:** 2025-12-06

**Authors:** Hironobu Sasaki, Kazuma Yagi, Yoko Kuga, Haruki Katahira

**Affiliations:** 1 Department of Internal Medicine, Sainokuni Higashiomiya Medical Center, Saitama, JPN; 2 Department of Surgery, Sainokuni Higashiomiya Medical Center, Saitama, JPN; 3 Department of Ophthalmology, Sainokuni Higashiomiya Medical Center, Saitama, JPN

**Keywords:** central diabetes insipidus, endocrine evaluation, hormone replacement therapy, lymphocytic hypophysitis, pituitary stalk enlargement, progressive pituitary dysfunction, stalk effect

## Abstract

Lymphocytic hypophysitis (LYH) is a rare autoimmune inflammatory disorder of the pituitary gland that can involve either the anterior or posterior lobe, or both. In some cases, it initially presents with isolated central diabetes insipidus (CDI) and later progresses to anterior pituitary hormone deficiencies. However, longitudinal descriptions of this progression are limited. A 66-year-old woman presented with dry mouth, polydipsia, and polyuria. Magnetic resonance imaging (MRI) revealed symmetrical pituitary enlargement with loss of the posterior pituitary bright spot, thickening of the pituitary stalk, and homogeneous contrast enhancement of both the pituitary stalk and posterior pituitary. Hormonal testing confirmed preserved anterior pituitary function but impaired vasopressin secretion, consistent with CDI secondary to lymphocytic posterior hypophysitis. During follow-up, serum prolactin levels gradually increased, while cortisol and thyroid hormone levels declined. Approximately one year after the onset of symptoms, MRI showed further pituitary stalk enlargement, and stimulation tests demonstrated diminished responses of adrenocorticotropic hormone, cortisol, and luteinizing hormone. Combined anterior pituitary hormone deficiencies were diagnosed, and replacement therapy with hydrocortisone and levothyroxine was initiated, resulting in symptomatic improvement. Serial hormonal and radiologic assessments revealed the progression from isolated posterior pituitary dysfunction to combined anterior pituitary failure, accompanied by pituitary stalk enlargement.

These findings suggest that both mechanical compression resulting from pituitary stalk enlargement and direct inflammatory extension from the posterior pituitary may contribute to the development of anterior pituitary dysfunction. This case was considered to represent LYH, showing possible dynamic changes in pituitary function and morphology over time. Administration of pharmacologic-dose steroids or pituitary biopsy should be considered if further progressive pituitary enlargement or new mass effects, such as headache or visual disturbance, become evident.

## Introduction

Lymphocytic hypophysitis (LYH) is a rare autoimmune inflammatory disorder of the pituitary gland, characterized by lymphocytic and plasma cell infiltration, leading to glandular enlargement and varying degrees of pituitary hormone deficiency [1]. The clinical manifestations depend on the regions of the pituitary affected. Enlargement of the pituitary can cause headache and visual disturbance, and impairment of anterior pituitary function may lead to fatigue, weakness, anorexia, weight loss, amenorrhea, or hypothyroid-like symptoms secondary to adrenal or thyroid insufficiency. In contrast, posterior pituitary involvement typically manifests as polyuria and polydipsia resulting from central diabetes insipidus (CDI) [2]. The diagnosis of LYH is often presumptive, relying on a combination of clinical, biochemical, and radiologic features [3], as definitive histologic confirmation requires pituitary biopsy, which is rarely performed due to its invasiveness [4]. Magnetic resonance imaging (MRI) plays a crucial role in the radiologic assessment of LYH, typically showing symmetrical pituitary enlargement, homogeneous contrast enhancement, thickening of the pituitary stalk, and loss of the posterior pituitary bright spot in cases with CDI [5].

These findings, in conjunction with the exclusion of secondary causes such as tumors, infections, and systemic inflammatory diseases, support a presumptive diagnosis of LYH [6]. In some cases, LYH initially presents with isolated posterior pituitary involvement, manifesting as CDI, and subsequently extends to the anterior pituitary, leading to combined pituitary hormone deficiency or panhypopituitarism. The interval between posterior pituitary involvement and the development of anterior pituitary dysfunction varies widely, ranging from one month to several years [7,8]. However, the precise mechanism underlying this progression remains unclear, and longitudinal descriptions evaluating serial endocrine and radiologic changes are limited. Here, we report a case considered to represent LYH that notably provides a longitudinal perspective, illustrating serial endocrine-radiologic correlations during the progression from isolated posterior pituitary dysfunction to combined anterior pituitary hormone deficiencies over a one-year period, accompanied by progressive pituitary stalk enlargement.

## Case presentation

**Figure 1:**
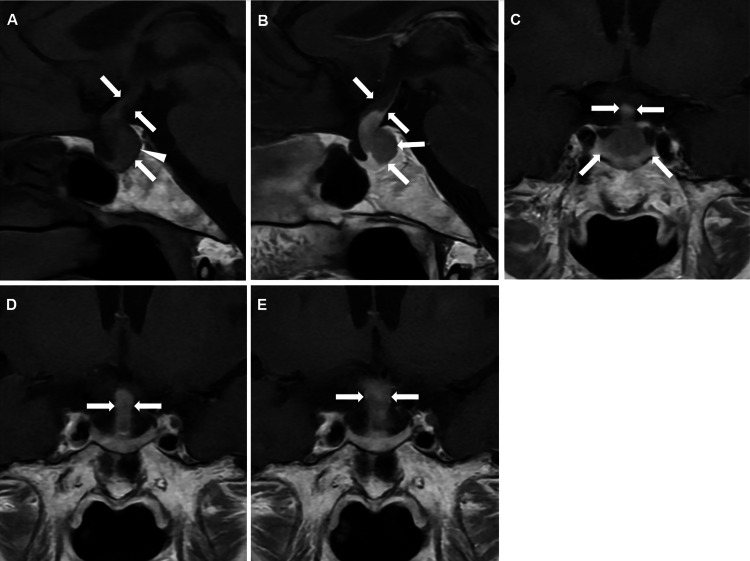
Pituitary magnetic resonance imaging (MRI) findings. MRI findings at presentation (A-D) and 234 days after the first admission (E). Non-contrast sagittal T1-weighted image (A), contrast-enhanced sagittal T1-weighted image (B), and contrast-enhanced coronal T1-weighted images (C-E). A mass-like lesion is seen extending from the pituitary gland to the pituitary stalk within the sella turcica. The normal high signal intensity of the posterior pituitary on T1-weighted imaging is absent (white arrows) (A). The pituitary stalk and posterior pituitary show homogeneous enhancement on the contrast-enhanced study, suggesting lymphocytic posterior hypophysitis (white arrows) (B). There is no evidence of compression of the optic chiasm by the pituitary gland (white arrows) (C). Compared with the initial MRI (white arrows) (D), further enlargement of the pituitary stalk is observed on the follow-up MRI (white arrows) (E).

A 66-year-old Japanese woman weighing 42.2 kg (BMI 19.5 kg/m^2^) presented with dry mouth, excessive thirst requiring approximately six liters of water per day, and polyuria. These symptoms had developed approximately four months prior to hospitalization. One month before admission, the patient visited a previous physician; however, laboratory tests ruled out diabetes mellitus. Given the persistence of her symptoms, diabetes insipidus was suspected, and the patient was referred to our institution for further evaluation. The patient had a history of right-sided breast cancer but was not taking any medications at the time of presentation. MRI revealed a mass-like lesion extending from the pituitary gland to the pituitary stalk within the sella turcica. On non-contrast T1-weighted images, the normal high-intensity signal of the posterior pituitary was absent (Figure 1A). Contrast-enhanced images demonstrated relatively homogeneous enhancement involving the pituitary stalk and posterior pituitary (Figure 1B). These findings suggested CDI secondary to lymphocytic posterior hypophysitis; therefore, the patient was hospitalized for further investigation, including hormonal assessments and stimulation tests.

The differential diagnoses of secondary hypophysitis include immunoglobulin G4 (IgG4)-related hypophysitis, antineutrophil cytoplasmic antibody (ANCA)-related hypophysitis, malignant lymphoma, sarcoidosis, tuberculosis, syphilis, fungal infection, germinoma, and immune-related adverse events. On admission, serum levels of IgG4, ANCA, soluble interleukin-2 receptor, angiotensin-converting enzyme, T-SPOT, rapid plasma reagin, treponema pallidum latex agglutination, β-D-glucan, α-fetoprotein, and human chorionic gonadotropin β-subunit were measured. However, all values were within or below the reference range. The laboratory data obtained on admission are summarized in Table 1.

**Table 1:** Laboratory data on the first admission. ACE: angiotensin-converting enzyme; ACTH: adrenocorticotropic hormone; AFP: alpha-fetoprotein; Alb: albumin; ALP: alkaline phosphatase; ALT: alanine aminotransferase; AST: aspartate aminotransferase; AVP: arginine vasopressin; BUN: blood urea nitrogen; Ca: calcium; CK: creatine kinase; Cl: chloride; Cr: creatinine; CRP: C-reactive protein; DHEA-S: dehydroepiandrosterone sulfate; Eo: eosinophil; FSH: follicle-stimulating hormone; FT3: free triiodothyronine; FT4: free thyroxine; γ-GTP: gamma-glutamyl transferase; GH: growth hormone; Hb: hemoglobin; HbA1c: glycated hemoglobin A1c; hCG-β: human chorionic gonadotropin beta-subunit; Ht: hematocrit; IGF-1: insulin-like growth factor-1; IgG: immunoglobulin G; K: potassium; LDH: lactate dehydrogenase; LH: luteinizing hormone; Mg: magnesium; MPO-ANCA: myeloperoxidase-specific antineutrophil cytoplasmic antibody; Na: sodium; P: phosphorus; Plt: platelet; PR3-ANCA: proteinase 3-specific antineutrophil cytoplasmic antibody; PRL: prolactin; RBC: red blood cell; RPR: rapid plasma reagin; sIL-2R: soluble interleukin-2 receptor; T-Bil: total bilirubin; TP: total protein; TPLA: treponema pallidum latex agglutination; TSH: thyroid-stimulating hormone; UA: uric acid; WBC: white blood cell

Test	Result	Reference range
Blood biochemistry		
TP (g/dL)	8.5	6.6-8.1
Alb (g/dL)	4.6	4.1-5.1
T-Bil (mg/dL)	0.5	0.4-1.5
AST (U/L)	22	13-30
ALT (U/L)	14	7-23
LDH (U/L)	245	124-222
ALP (U/L)	59	38-113
γ-GTP (U/L)	25	9-32
CK (U/L)	244	41-153
BUN (mg/dL)	11.5	8-20
Cr (mg/dL)	0.63	0.46-0.79
UA (mg/dL)	6.8	2.6-5.5
Na (mEq/L)	145	138-145
K (mEq/L)	3.7	3.6-4.8
Cl (mEq/L)	108	101-108
Ca (mg/dL)	9.8	8.8-10.1
P (mg/dL)	3.4	2.7-4.6
Mg (mg/dL)	2.6	1.8-2.6
CRP (mg/dL)	0.03	0-0.14
Glucose (mg/dL)	91	73-109
HbA1c (%)	5.6	4.9-6.0
Peripheral blood		
WBC (/mm^3^)	5000	3300-8600
Eo (%)	4	0-10
RBC (×10^4^/mm^3^)	415	386-492
Hb (g/dL)	13.3	11.6-14.8
Ht (%)	40.6	35.1-44.4
Plt (×10^4^/mm^3^)	25.6	15.8-34.8
Endocrine evaluation		
GH (ng/mL)	0.53	0.13-9.88
IGF-1 (ng/mL)	69	12-798
PRL (ng/mL)	20.0	6.1-30.5
LH (mIU/mL)	6.0	1.1-88.3
FSH (mIU/mL)	30.3	1.5-157.8
Estradiol (pg/mL)	<10.0	<47.0
TSH (µIU/mL)	2.36	0.38-5.38
FT3 (pg/mL)	2.94	1.68-3.67
FT4 (ng/dL)	1.02	0.70-1.48
ACTH (pg/mL)	28.5	7.2-63.3
Cortisol (µg/dL)	16.4	7.07-19.6
DHEA-S (µg/dL)	70	12-133
AVP (pg/mL)	0.4	<2.8
Etiological evaluation of hypophysitis		
IgG (mg/dL)	1822	861-1747
IgG4 (mg/dL)	69	11-121
PR3-ANCA (U/mL)	<1.0	0-3.5
MPO-ANCA (U/mL)	<1.0	0-3.5
sIL-2R (U/mL)	217	122-496
ACE (U/L)	6.0	7.0-25.0
T-SPOT	Negative	-
RPR	Negative	-
TPLA	Negative	-
β-D-glucan (pg/mL)	3.86	<11.00
AFP (ng/mL)	2.6	0-10.0
hCG-β (ng/mL)	<1.0	<1.0

**Figure 2:**
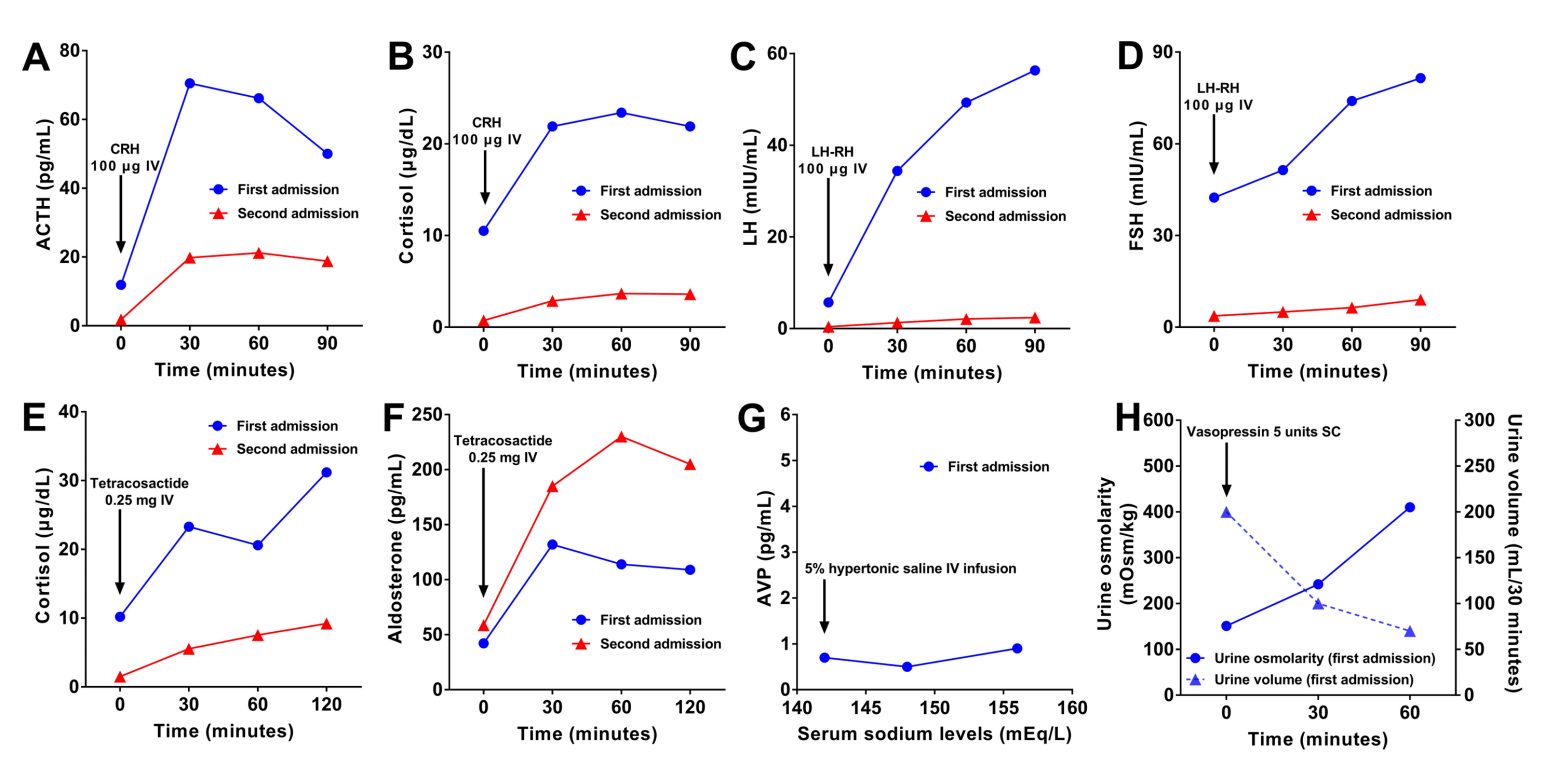
Comparison of stimulation test results between the first and second admissions. Responses of ACTH (A) and cortisol (B) to the CRH stimulation test; LH (C) and FSH (D) to the LH-RH stimulation test; and cortisol (E) and aldosterone (F) to the rapid ACTH stimulation test. In the CRH and LH-RH stimulation tests (A-D), the interval between the first and second tests was 253 days, whereas in the rapid ACTH stimulation test (E-F), the interval was 252 days. The hypertonic saline infusion test (G) and the vasopressin stimulation test (H) were performed only during the first admission. ACTH: adrenocorticotropic hormone; AVP: arginine vasopressin; CRH: corticotropin-releasing hormone; FSH: follicle-stimulating hormone; IV: intravenous; LH: luteinizing hormone; LH-RH: luteinizing hormone-releasing hormone; SC: subcutaneous injection

The patient had no history of immune checkpoint inhibitor therapy, and whole-body contrast-enhanced computed tomography revealed no organ enlargement or other findings suggestive of IgG4-related disease. During a 24-hour urine collection, urinary cortisol excretion was 53.9 µg/day, indicating adequate cortisol secretion. However, the 24-hour urine volume was elevated at 4900 mL, and the urine osmolality was low at 121 mOsm/kg. The corticotropin-releasing hormone (CRH) and rapid adrenocorticotropic hormone (ACTH) stimulation tests demonstrated appropriate increases in ACTH and cortisol (Figures 2A, 2B, 2E). In addition, the luteinizing hormone-releasing hormone (LH-RH) stimulation test elicited adequate responses of luteinizing hormone (LH) and follicle-stimulating hormone (FSH) (Figures 2C-2D). During the 5% hypertonic saline infusion test, arginine vasopressin (AVP) secretion in response to rising serum sodium was minimal (Figure 2G). In the vasopressin stimulation test, the patient showed an appropriate response, with a subsequent decrease in urine volume and an increase in urine osmolality (Figure 2H), supporting a diagnosis of CDI. Based on these findings, the patient was suspected to have CDI, likely secondary to lymphocytic posterior hypophysitis, with preserved anterior pituitary function. Desmopressin orally disintegrating tablets (60 μg/day) were initiated, and outpatient follow-up was planned. The patient had no headache, and ophthalmologic examination revealed no visual field defects. Furthermore, MRI showed no evidence of the optic chiasm compression caused by the pituitary lesion (Figure 1C). Given the absence of mass effect or compressive symptoms, pharmacologic doses of steroids were not administered.

The changes in hormone levels during follow-up are shown in Table 2. All hormone measurements were obtained from blood samples collected around 1:00 p.m. Prolactin levels gradually increased over time, whereas thyroid hormone and cortisol levels declined. Despite these hormonal changes, fluorodeoxyglucose-positron emission tomography performed 52 days after the initial hospitalization showed no evidence of pituitary metastasis from breast cancer. At the outpatient visit 215 days after the first hospitalization, the patient reported severe fatigue and nausea. Pituitary MRI performed 234 days after the initial admission revealed no interval change in the signal intensity, size, or contrast enhancement of the pituitary gland compared with the baseline study. In contrast, the pituitary stalk demonstrated enlargement, with its maximal diameter increasing from 5.17 mm to 7.59 mm (Figures 1D-1E). Considering the possibility that progressive pituitary stalk enlargement had led to compression-induced anterior pituitary dysfunction, hydrocortisone (15 mg/day) was initiated, followed by the addition of levothyroxine (50 µg/day). Although there was no worsening of headache or visual field impairment, the patient was subsequently readmitted for reassessment of pituitary hormonal function.

**Table 2:** Changes in hormone levels after discharge following the first admission. X indicates the date of the first admission. All four blood samples listed in the table were obtained around 1:00 p.m. ACTH: adrenocorticotropic hormone; DHEA-S: dehydroepiandrosterone sulfate; FT3: free triiodothyronine; FT4: free thyroxine; GH: growth hormone; IGF-1: insulin-like growth factor-1; PRL: prolactin; TSH: thyroid-stimulating hormone

Test	X+61 (days)	X+89 (days)	X+131 (days)	X+215 (days)	Reference range
GH (ng/mL)	0.15	0.29	0.24	0.09	0.13-9.88
IGF-1 (ng/mL)	76	64	64	48	12-798
PRL (ng/mL)	17.1	38.5	65.2	96.4	6.1-30.5
TSH (µIU/mL)	1.12	1.24	1.41	2.69	0.38-5.38
FT3 (pg/mL)	2.29	2.21	2.35	2.19	1.68-3.67
FT4 (ng/dL)	0.85	0.83	0.76	0.66	0.70-1.48
ACTH (pg/mL)	7.3	12.9	10.2	9.5	7.2-63.3
Cortisol (µg/dL)	7.70	6.38	5.33	3.58	7.07-19.6
DHEA-S (µg/dL)	46	48	44	22	12-133

At the time of readmission, the basal levels of ACTH, cortisol, LH, and FSH were lower than those observed at the first admission. In addition, the hormonal responses to the CRH, LH-RH, and rapid ACTH stimulation tests were all attenuated (Figures 2A-2C, 2E), except for FSH, which exhibited a slightly reduced but still normal response (Figure 2D), and aldosterone, which exhibited an exaggerated response compared with the first admission (Figure 2F). After initiation of hydrocortisone therapy, the patient reported improvement in fatigue and nausea, while worsening polyuria, with a daily urine output of 3300 mL; therefore, the dose of desmopressin was increased to 120 µg/day.

## Discussion

In our case, at the time of the first admission, anterior pituitary function was preserved as confirmed by stimulation tests. Based on MRI findings and the results of the hypertonic saline infusion test, which showed impaired AVP secretion in response to rising serum sodium levels, together with a good response to the vasopressin stimulation test, the condition was considered to represent lymphocytic posterior hypophysitis presenting with CDI [9]. Adrenal insufficiency manifested seven months after the first admission, and symptoms of CDI had already been present four months prior to that admission. Therefore, we consider that anterior pituitary dysfunction developed approximately one year after the onset of posterior dysfunction. We were able to track hormonal changes and alterations in MRI findings over the course of the progression from posterior to anterior pituitary dysfunction, providing a longitudinal correlation between endocrine function and radiologic changes, and we confirmed hormonal responsiveness in stimulation tests. In contrast to prior reports that largely describe static, single-time-point observations, this case illustrates the dynamic evolution of LYH over time.

Previous reports have indicated that the interval between posterior and anterior pituitary involvement in LYH varies widely, ranging from a month to several years [7,8], although the underlying mechanism of this progression remains incompletely understood. In our case, several possible mechanisms could explain the progression of pituitary dysfunction. Enlargement of the pituitary stalk was observed in association with a decline in cortisol and thyroid hormone levels and a gradual increase in serum prolactin. Enlargement or inflammation of the pituitary stalk can compromise the hypothalamic-pituitary portal circulation, interrupting the transport of releasing and inhibiting hormones from the hypothalamus to the anterior pituitary [10,11]. This may lead to secondary hypopituitarism due to reduced hypothalamic stimulation and, consequently, decreased secretion of ACTH, LH, and FSH [10]. Among these hormonal changes, mild hyperprolactinemia is a well-recognized finding and is attributed to impaired dopaminergic inhibition of lactotrophs, a phenomenon known as the “stalk effect.” Inflammatory involvement of the pituitary stalk can reduce dopamine delivery, resulting in prolactin elevation [11,12]. The concurrent decline in anterior pituitary hormones, cortisol, and thyroid hormone concentrations, and the rise in prolactin observed in our patient support the hypothesis that pituitary stalk inflammation disrupted hypothalamic-pituitary signaling, leading to the progression from isolated posterior pituitary dysfunction to involvement of the anterior pituitary. In suprapituitary lesions, the ACTH response to CRH stimulation is typically delayed and exaggerated during the early phase [13]. However, at the second admission, the ACTH response to CRH stimulation was blunted. Although chronic disruption of the pituitary stalk can lead to diminished ACTH responsiveness due to long-standing loss of hypothalamic input, the transition from a normal to a blunted response within only eight months suggests progressive impairment of the anterior pituitary itself. Considering that LH also showed a reduced response to LH-RH stimulation during this period, not only mechanical compression of the pituitary stalk but also direct inflammatory involvement extending from the posterior to the anterior lobe should be considered. No interval changes in the pituitary gland were observed on MRI compared with the initial imaging. However, since the absence of MRI changes does not rule out hypophysitis [14], clinical and endocrine evaluations should be prioritized.

Given the present case’s progression from isolated posterior pituitary dysfunction to combined anterior and posterior pituitary dysfunction within approximately one year, determining the optimal timing of treatment becomes critically important. According to a recent meta-analysis, initial high-dose glucocorticoid (GC) therapy was associated with significantly higher odds of anterior pituitary hormone recovery compared with observation alone [15]. However, the same analysis also reported that the need for additional treatment, such as repeat courses of high-dose GCs, surgical intervention, alternative immunosuppressive agents (including methotrexate, azathioprine, or rituximab), or radiotherapy, was markedly higher among patients who initially received high-dose GC therapy [15]. Furthermore, the recovery rate of CDI did not differ significantly between patients treated with high-dose GCs and those managed conservatively [15]. Considering the wide range of potential adverse effects associated with high-dose GCs, their initiation should be carefully considered, taking into account the balance between risks and benefits. Some authors have suggested that high-dose GC therapy should be initiated when a significant mass effect is observed during follow-up [3]. The patient’s overall condition remains stable under hormone replacement therapy, and this conservative management was considered appropriate given the absence of a clinically significant mass effect, the diagnostic uncertainty due to the lack of histopathological confirmation, and the potential risk of overtreatment associated with high-dose GCs. However, pharmacologic-dose steroid therapy should be initiated if headache or visual disturbances develop.

The main limitation of this case is the absence of a pathological diagnosis obtained through pituitary biopsy. The diagnosis remains presumptive, based on hormonal deficiencies, pituitary enlargement with homogeneous contrast enhancement on MRI, and the exclusion of other potential etiologies through laboratory testing. Therefore, the possibility of coexistence of other inflammatory or neoplastic disorders cannot be completely ruled out. Measurement of anti-rabphilin-3A antibody, a known marker of lymphocytic infundibuloneurohypophysitis [16], could not be performed because the assay was unavailable at our institution. Although a pituitary biopsy is required for a definitive diagnosis, it carries a risk of inducing new pituitary dysfunction and should therefore be approached with caution [17]. However, when pathological confirmation is essential for therapeutic decision-making, such as initiating long-term pharmacologic-dose steroid treatment, a biopsy may be warranted [3]. Accordingly, treatment strategies should be determined based on a comprehensive assessment of the clinical course, imaging findings, and endocrinological results, with vigilant follow-up even in the absence of pathological confirmation.

## Conclusions

This case enabled serial hormonal assessments and MRI follow-up, which clearly demonstrated the transition from CDI to combined anterior pituitary hormone deficiencies, accompanied by progressive pituitary stalk enlargement. These findings suggest that mechanical compression caused by pituitary stalk enlargement may have contributed to the progression of pituitary dysfunction, while direct inflammatory extension from the posterior to the anterior lobe could also have played a role. Although this case was managed conservatively, the timely initiation of pharmacologic-dose steroid administration and consideration of a preceding pituitary biopsy may be warranted when further progressive pituitary enlargement or mass effects, including headache or visual impairment, become evident. This case highlights the importance of individualized treatment decisions that balance diagnostic certainty, disease activity, and potential treatment risks.
